# Effects of Food Additives on Immune Cells As Contributors to Body Weight Gain and Immune-Mediated Metabolic Dysregulation

**DOI:** 10.3389/fimmu.2017.01478

**Published:** 2017-11-06

**Authors:** Heitor A. Paula Neto, Priscila Ausina, Lilian S. Gomez, João G. B. Leandro, Patricia Zancan, Mauro Sola-Penna

**Affiliations:** ^1^Laboratório de Alvos Moleculares, Faculdade de Farmácia, Departamento de Biotecnologia Farmacêutica, Universidade Federal do Rio de Janeiro, Rio de Janeiro, Brazil; ^2^Laboratório de Enzimologia e Controle do Metabolismo, Faculdade de Farmácia, Departamento de Biotecnologia Farmacêutica, Universidade Federal do Rio de Janeiro, Rio de Janeiro, Brazil; ^3^Laboratório de Oncobiologia Molecular, Faculdade de Farmácia, Departamento de Biotecnologia Farmacêutica, Universidade Federal do Rio de Janeiro, Rio de Janeiro, Brazil

**Keywords:** food additives, type 2 diabetes, metabolic syndrome, metainflammation, microbiota, citrate, immunometabolism

## Abstract

Food additives are compounds used in order to improve food palatability, texture, and shelf life. Despite a significant effort to assure safety of use, toxicological analysis of these substances, generally, rely on their direct toxicity to target organs (liver and kidney) or their genotoxic effects. Much less attention is paid to the effects of these compounds on cells of the immune system. This is of relevance given that metabolic dysregulation and obesity have a strong immune-mediated component. Obese individuals present a state of chronic low-grade inflammation that contributes to the establishment of insulin resistance and other metabolic abnormalities known as the metabolic syndrome. Obesity and metabolic syndrome are currently recognized as worldwide epidemics that pose a profound socioeconomic impact and represent a concern to public health. Cells of the immune system contribute to both the maintenance of “lean homeostasis” and the metabolic dysregulation observed in obese individuals. Although much attention has been drawn in the past decades to obesity and metabolic syndrome as a result of ingesting highly processed food containing large amounts of fat and simple sugars, mounting evidence suggest that food additives may also be important contributors to metabolic derangement. Herein, we review pieces of evidence from the literature showing that food additives have relevant effects on cells of the immune system that could contribute to immune-mediated metabolic dysregulation. Considering their potential to predispose individuals to develop obesity and metabolic syndrome, their use should be taken with caution or maybe revisited.

## Introduction

### Obesity and Metabolic Syndrome

Obesity has reached worldwide epidemic proportions in the last decades. This is accompanied by an increased prevalence of comorbidities, such as type II diabetes, cardiovascular diseases, cancer, and other chronic diseases. In fact, 7 out of 10 leading causes of death in the U.S. are chronic disease states of which many have associations with obesity (1–3). Altogether, obesity and its comorbidities impose high social and economic costs to individuals and society alike. Therefore, understanding the causes of obesity and designing preventive strategies are fundamental demands.

The factors that contribute to obesity are many (2), but it is generally accepted that obesity results from an association of genetic predisposition (which is currently unmodifiable) with a dysregulated energy balance. The World Health Organization (WHO) defines obesity as a body mass index (BMI, weight in kilograms divided by the square of height in meters) greater or equal to 30 kg/m^2^ (3). Based on this definition, obesity can be considered a disorder of disproportionate mass of an individual. In that sense, it is reasonable to think of obesity as a result of positive energy balance, that is, increased calorie intake (especially in the form of dense calorie processed food) and decreased calorie expenditure (sedentary lifestyle).

Based on the positive energy balance view, excessive calorie intake and sedentary habits are considered the main modifiable factors contributing to the obesity epidemic. Special attention has been drawn to the often consumed highly processed and energy-dense foods. In fact, many obesity models in experimental animals rely on feeding mice and rats a high-fat or a high-fat, high-sugar diet. Therefore, the sugar and lipid content of foods and drinks are always taken into account when referring to weight gain and obesity.

### Metainflammation and Insulin Resistance

The concept of obesity as excessive weight due to increased fat deposition is simplistic. Over the last two decades, cumulating pieces of evidence support the concept that fat tissue hyperplasia and hypertrophy is accompanied by profound alterations in adipose tissue homeostasis. The breakthrough description of elevated tumor necrosis factor α (TNFα) levels in adipose tissue of obese individuals and the contribution of TNFα to the establishment of insulin resistance (4) paved the way for the concept of an inflammatory component of obesity. This inflammatory component is referred to as “metainflammation,” a pathological condition of chronic, low-grade inflammation observed in obese subjects (5, 6).

Later studies have shown macrophage infiltration in obese fat tissue and their contribution to pro-inflammatory cytokine production (including TNFα) and insulin resistance. More recently, studies have shown the participation of many immune cell types in adipose tissue homeostasis, dysregulation, and an important contribution of macrophage polarization. In lean individuals, adipose tissue has a prevalence of alternatively activated (or M2) macrophages. These M2 macrophages produce anti-inflammatory cytokines, such as IL-10, and express arginase-1, which metabolizes arginine to polyamines and away from free radical nitric oxide (NO). Various immune cell types maintain M2 polarization in lean adipose tissue; T cells (Tregs) that contribute to leanness (7) and may be a relevant source of IL-10, a cytokine that polarizes macrophages to a M2 phenotype in adipose tissue (8), and eosinophils, which are the main source of the M2-polarizing cytokine IL-4 in adipose tissues (9). These cytokines and M2 macrophages sustain adipose tissue homeostasis and contribute to insulin sensitivity.

In obese adipose tissue, on the other hand, pro-inflammatory mediators such as TNFα and the CC chemokine ligand C–C motif ligand 2 recruit blood monocytes, where these cells become polarized toward the “classical” pro-inflammatory M1 state (10, 11). Interestingly an important contributor to M1 polarization is the activation of TLR4 by free fatty acids (12), which are found in large amounts due to increased lipolysis in obese adipose tissue. In contrast to their M2 counterparts, M1 macrophages produce pro-inflammatory mediators, such as TNF-α, IL-1β, and leukotriene B_4_, and express the inducible isoform of NO synthase (iNOS), leading to the production of large amounts of NO from arginine. Altogether, these mediators contribute to adipose tissue insulin resistance and, consequently, a sustained state of lipolysis which contributes to the perpetuation of the local low-grade inflammatory state. Moreover, eosinophil and Treg numbers were shown to lower in obese adipose tissue further favoring M1 polarization.

A macrophage M1–M2 imbalance can also be seen in other target organs, such as the liver (13). In lean individuals, liver-resident macrophages, known as Kupffer cells (KCs) show a M2-like phenotype, sustained by their PPARδ expression (14). Mediators derived from obese adipose tissue, especially TNF-α and FFAs are able to polarize KCs to a M1-like state. This is further complicated by the fact that M1-KCs produce mediators that recruit M1-prone monocytes from the circulation to populate the liver (15). This process is directly related to the establishment of liver insulin resistance and the progress of liver steatosis to cirrhosis and liver failure. In fact, depletion of liver macrophages prevents steatohepatitis and insulin resistance in mice (16, 17). Besides endogenous mediators, KCs can be activated by microbial products derived from the gut (18). The liver is anatomically positioned so that it is the first organ to contact gut-derived products. Actually, this is one proposed mechanism by which disturbances in gut barrier—either directly or through an imbalance of the gut microbiome—can result in liver inflammation and metabolic derangements (18).

In summary, the current view is that the modern lifestyle, characterized by overeating associated with low physical activity, leads to increased weight and fat mass gain through a shifted energy balance. This disturbs the homeostasis of target organs, such as the liver and adipose tissue, likely through mechanisms involving the pro-inflammatory activation of immune cells, leading to peripheral insulin resistance. In the longer term, these alterations will culminate in metabolic syndrome, characterized by abdominal fat, hypertension, dyslipidemia, insulin resistance, and steatohepatitis.

### Food Additives As Neglected Players in Obesity and Metabolic Syndrome

In modern societies, the easy access to food, together with the demand for highly palatable and ready-to-serve products lead to the generalized consumption of industrialized processed foods. Processed food lacking in vitamins, fibers, and minerals, and dense in calories in the form of fat and simple sugar are, thus, considered the main villains of modern diets (19). The contribution of excessive calorie intake to the alarming increase in obesity witnessed in the last three decades is undeniable.

However, processed food is composed of not only sugar and fat but also a series of other products that are added in order to increase palatability, modify texture, and prolong shelf life. These products are collectively called food additives. A few of these additives even have well-known beneficial health effects as exemplified by probiotics and prebiotics. These additives are used to directly or indirectly influence gut microbiota and have well-documented benefits for host health and well-being (20). All prospected food additives, however, need to pass a rigorous scrutiny for their potential toxicity and undesired side effects. These toxicity tests are carried out following a series of guidelines, including outcome parameters that must be observed. These parameters comprise analysis of clinical manifestations, biochemical and hematological alterations, and postmortem analysis (21, 22). Based on these results, food additives are allowed to be used in varying amounts and many of them are considered innocuous and safe. However, studies sometimes present evidence that questions food additive safety and asks for a reassessment of their use. In this regard, we present evidence from the current literature that food additives—even some that are generally considered safe—may have relevant effects on immune cells and, thus, could also contribute to the burden of obesity and its related comorbidities. We have selected a few examples to illustrate and base a discussion on effects that food additives may have on cells of the immune system, which may potentially contribute to a series of pathologic and metabolic conditions. These examples are summarized in Table 1 and discussed in detail in the subsequent sections.

**Table 1:** Common food additives and their proposed effects on cells of the immune system and metabolic parameters.

Additive	Max. daily intake (FDA)	Described effects	Reference
Sucralose	5 mg/kg	Dysbiosis in rats Dysbiosis and impaired glucose tolerance in mice	(23) (24)
Saccharin	15 mg/kg	Dysbiosis and impaired glucose tolerance in mice	(24)
Aspartame	50 mg/kg	Impaired glucose tolerance	(25, 26)
Carboxymethylcellulose	No limitations	Weight gain Impaired glucose tolerance	(27)
Polysorbate-80	25 mg/kg	Low-grade inflammation increased adiposity	(27)
Citrate	No limitations	Increased fasting glycemia and impaired glucose tolerance Potentiate LPS-induced activation of macrophage cell line (THP-1)	(28) (29)
Sodium	2,400 mg/day	Exacerbates TNBS-induced colitis in mice Favors M1 macrophage polarization Favors Th17 polarization and predispose mice to develop autoimmune disease	(30) (31) (32, 33)
Carrageenan	No limitations	Glucose intolerance in mice Exacerbated glucose intolerance and dyslipidemia induced by HFD in mice	(34) (35)

*Food additives, their described effects, and their maximum daily intake recommendations established by Food and Drug Administration*.

## When “Natural” may Not be Healthy: The Case of Citrate

Citrate is a very common food and drink additive widely used by the food industry as a chemical acidifier, flavoring agent and a preservative (36, 37). Since it is found in large amounts in many fruits and vegetables, especially citric fruits, it is generally considered natural and healthy. The Food and Drug Administration (FDA), for example, does not pose a limit for citric acid addition to foods or drinks.

Apart from being a food additive, citrate is also a metabolite involved in carbohydrate and lipid metabolisms. In situations of anabolism, as after meals, citrate is formed in the mitochondria and exported to the cytosol. Cytosolic citrate is metabolized to acetyl-CoA and oxaloacetate by the enzyme ATP:citrate lyase (ACLY) (38). Cytosolic acetyl-CoA then serves as the main building block for fatty acids and cholesterol synthesis. Despite the well-described fate of endogenous citrate, the fate of ingested citrate remains largely unexplored. Citrate enters the cells through the SLC13A5 citrate transporter encoded by the mINDY (mammalian I’m Not Dead Yet) gene (39). In humans, this gene is primarily expressed in the liver, presenting very low but detectable expression in the brain, testes, and ovary (40). Moreover, expression of this citrate transporter is upregulated in the liver of animals submitted to a high-fat, high-sucrose diet (40). Since ACLY is also highly expressed in the liver (41), it is reasonable to assume that food-derived citrate may contribute to postprandial lipid and cholesterol synthesis. Although expression of SLC13A5 in inflammatory cells such as monocytes, has not been reported, it has been reported that exogenous citrate modulates liposaccharide-induced monocyte inflammatory responses in cell culture (29), raising the question of the role of exogenous citrate in inflammation.

We recently addressed this issue by supplementing citrate in the drinking water of mice fed a standard diet (28). Contrary to our expectations, we did not find any increase in weight gain, adipose tissue mass, ectopic lipid deposition, or plasmatic lipid levels. This suggests that citrate supplementation did not contribute significantly to *de novo* lipid synthesis, at least in the medium-term duration (75 days) of our experiment. We currently do not know how citrate supplementation may affect lipid metabolism in the long term. On the other hand, we found a significant effect on glucose homeostasis. Mice receiving citrate plus sucrose showed higher fasting glycemia and diminished glucose tolerance. This was accompanied by increased levels of inflammatory cytokines in adipose tissue (28).

The mechanisms by which exogenous citrate may contribute to adipose tissue inflammation and establishment of insulin resistance remains to be determined. In this regard, recent data suggest that citrate may be pro- or anti-inflammatory. For example, citrate administration was shown to protect mice from cerebral and hepatic oxidative damage induced by low-dose LPS (42). Parameters of tissue damage, such as DNA fragmentation and hepatic enzymes in plasma, were significantly diminished in citrate-treated mice. However, this was only observed at lower citrate doses. At the highest dose tested citrate showed no effect or even a potentiating effect. *In vitro*, exogenous citrate was recently shown to potentiate LPS-induced activation of THP-1 monocyte cell line (29). This was dependent on calcium availability, since higher citrate concentrations showed an opposite inhibitory effect that was rescued by calcium supplementation. Potentiation of LPS effects by citrate could be blocked by TCA, an ACLY competitive inhibitor, suggesting the participation of cytosolic citrate metabolism in this effect. Indeed, cytosolic citrate accumulation is a metabolic characteristic of inflammatory macrophages (43) and some effector functions of macrophages depend on citrate metabolism (44). It was recently demonstrated that ACLY is important for macrophage synthesis of lipid mediators and free radical production. Interfering with ACLY expression or activity significantly inhibited prostaglandin synthesis and NO and reactive oxygen species production by LPS- or cytokine-activated macrophage cell lines (45). Moreover, mitochondrial citrate carrier and ACLY expression is upregulated by LPS in a NF-κB-dependent fashion, increasing citrate influx to the cytosol (46).

Furthermore, citrate may also have epigenetic effects on macrophages. ACLY plays a role in epigenetic regulation in diverse mammalian cell types. Histone acetylation is responsive to ACLY-dependent glucose availability (41, 47) and ACLY was shown to localize in the nucleus, where it can contribute to the epigenetic program of adipocyte differentiation as a source of acetyl groups for histone acetylation (48). In kidney mesangial cells, ACLY can also promote histone hyperacetylation and upregulation of fibrogenic genes, contributing to high glucose-triggered diabetic renal fibrosis (49). More specifically in macrophages, a recent report showed that epigenetic modifications underlying IL-4-mediated M2 polarization were dependent on ACLY activation triggered by Akt–mTOR pathway (50). Furthermore, citrate carrier acetylation and inhibition strongly decreases LPS-induced inflammatory response (46).

Finally, the contribution of citrate to obesity and its related comorbidities can be inferred indirectly by pharmacological data. Bempedoic acid, a lipid-regulating compound, was recently described as a potent ACLY inhibitor (51, 52). In mouse model of metabolic dysregulation, bempedoic acid treatment significantly reduced adiposity, plasma lipid levels, and attenuated inflammation onset and atherosclerotic lesion development (53). A phase-II clinical trial showed that bempedoic acid safely lowers LDL-c levels and could be used alone or in association with others therapies (51). Moreover, treatment with hydroxycitrate, which functions as ACLY inhibitor, attenuates weight gain, lipid deposition, and adipose tissue inflammation in spontaneous genetically obese rats (54).

These data support the hypothesis that citrate intake from foods and drinks may promote inflammation. Our results show that citrate contributes to adipose tissue inflammation and glucose intolerance even when mice are fed a regular diet. Although citrate effects may vary depending on experimental settings, i.e., may protect from LPS effects (42) or potentiate LPS effects (29), the abovementioned pieces of evidence suggest that it is important to reassess the concept that citrate is inert. Further studies are needed to address the metabolic effects of citrate supplementation in the long-term as well as in association with high-calorie diets.

## Salt: A View Beyond Hypertension

Salt, especially in the form of sodium chloride, is largely used worldwide to increase food flavor and palatability. High sodium intake correlates with the development of a series of diseases, being hypertension the most popularly known (55). Industrialized and processed foods usually contain high amounts of salt that can be up to 500 times more than a similar home-cooked meal (56). Moreover, associated with their high-sodium amounts, industrialized foods present low or undetectable levels of other minerals, especially those related to beneficial effects, such as magnesium, selenium, zinc, and others (57). The low consumption of these minerals is strongly associated with the prevalence of obesity, type 2 diabetes (T2D) (58), and inflammatory bowel disease (59, 60). The mechanisms underlying the detrimental effects of high salt consumption are still a matter of debate. However, recent data provide pieces of evidence that high salt may have a relevant impact on cells of the immune system.

Two independent studies have shown that a high-salt diet induces T cell polarization toward the pathogenic Th17 phenotype, predisposing mice to develop autoimmune disease (32, 33). This was shown to be dependent on the activation of the serine/threonine kinase serum glucocorticoid kinase (SGK)-1, p38-MAPK, and nuclear factor of activated T cells (NFAT) 5. Concerted activation of these factors leads to increased IL-23R expression and stabilization of the Th17 phenotype. Since Th17 cells play a pathogenic role in obesity and metabolic syndrome (61, 62), it is reasonable to assume that a similar mechanism may predispose individuals consuming large amounts of salt to develop metabolic derangements.

Similar to the findings in Th17 cells, macrophages also had their effector phenotype modulated by extracellular osmolarity (*in vitro*) and high salt consumption (*in vivo*). Through the same p38–NFAT5 pathway triggered in T cells, increased sodium concentrations were able to boost NO production and the leishmanicidal activity of macrophages (31). This suggests that high salt concentrations shift macrophage activation toward the M1 phenotype. In fact, high salt blunted M2 polarization induced by IL-4 and IL-13 and impaired their effector functions (63). This is interesting when considering, for example, the effects of citrate on macrophage activation. High sodium and citrate are commonly found in processed food and both seem to favor M1 polarization of macrophages. This could have profound influence on the development of insulin resistance and adipose tissue inflammation, especially if a synergic effect could be demonstrated in future studies. Moreover, a recent report described that macrophages are able to sense hyperosmotic stress and activate both the NLRP3 and NLRC4 inflammasomes (64). This was dependent on mitochondrial ROS production and lead to IL-1β secretion that contributed to a biased T cell polarization toward the Th17 phenotype. Given the well-documented participation of NLRP3 inflammasome and IL-1β in insulin resistance (65, 66), this could be a reasonable mechanism for the association between high salt consumption and the metabolic syndrome.

Finally, increased salt concentrations, either *in vitro* or *in vivo*, was shown to impair Treg differentiation and function (67). This effect was also dependent on SGK-1, resulted in loss of Treg suppressive function and increased interferon (IFN)-γ production, thus contributing to the aggravation of both graft-versus-host disease and experimental colitis. Taken together, these studies suggest that the consumption of a high-salt diet would strongly favor a pro-inflammatory immune response with heightened Th17 and M1 activation and decreased Treg and M2 functions. Given the well-documented pathological role of both Th17 T cells and M1 macrophages in obesity, it is reasonable to speculate that high salt consumption would favor obesity and diabetes by an immune-mediated mechanism involving a strong bias toward pro-inflammatory phenotype of both macrophages and T cells. In fact, high salt consumption has been associated with features of the metabolic syndrome, such as obesity, hypertension, and T2D (68). However, the impact of this salt-triggered pro-inflammatory bias to obesity and T2D remains to be determined. Moreover, the effect of chronic exposure to high salt diet has never been tested, neither in obesity nor in autoimmune models. Another open question is the outcome of high salt consumption in association with other additives with potential pro-inflammatory effects, such as citrate (above) or carrageenan and emulsifiers (discussed below). Nevertheless, the straight interrelationship among salt consumption, weight gain, and immune-associated metabolic dysregulation cannot be dismissed.

## The Gut Microbiome: A Symbiotic Relatioship with the Immune System Affecting Weight Gain and Type 2 Diabetes

The gut microbiota comprises a diverse community of microbes that inhabits the intestinal tract. It is now well demonstrated that the gut microbiota provides key signals for the full maturation of the immune system and also has important metabolic benefits for the host. On the other hand, disturbances of the microbiota–host relationship are associated with immune-mediated and metabolic-associated chronic diseases, such as diabetes and the metabolic syndrome (27). Mucus at the intestinal surface prevents direct contact between epithelial cells that line the intestine and the gut microbiota (69). Diverse agents (nutritional, chemical, or even an imbalance among the different bacteria species) that interfere with this multilayered mucus barrier might have the potential to promote or to control diseases associated with gut inflammation.

Regardless of the mucus barrier, gut microbiota interacts with immune cells, providing signals that are fundamental to the normal development of the host immune functions (70–72). Development of Th17 cells in the gut, for example, strictly depends on the presence of segmented filamentous bacteria (73, 74). By contrast, Clostridium and *Bacteroides fragilis* favor Tregs development (75, 76). In fact, any dysbiosis affecting the ileum microbiota deeply affect intestinal innate immunity and CD4 T cell homeostasis—particularly, a decrease in Th17 cells. These changes are the initial steps for the onset of metabolic diseases. Certainly, they are sufficient to induce glucose intolerance and insulin resistance.

In the gut, CD4 T cells contribute to immunity by differentiating into various subsets, notably inflammatory and regulatory cells (77, 78). Th17 cells are the most abundant CD4 T cells in mucosal tissues (79). They secrete two isoforms of IL-17 (IL-17A and IL-17F) and/or IL-22, which confer protection against fungi and pathogenic bacteria. Th17 cell differentiation is mediated by the transcription factor retinoid-related orphan receptor gamma t (80), induced by the cytokines TGFβ1, IL-6, IL-1β, and IL-21, and maintained by IL-23 (81).

It is remarkabe that 90% of the human gut microbiome is made up of organisms from two phyla, Firmicutes and Bacteroidetes. Importantly, an imbalance between these phyla has been directly linked to obesity, T2D, and inflammation (82, 83). Most of the studies report the augmentation in Firmicutes abundance and reduction in Bacteroidetes in obese versus lean individuals (84–87).

Corroborating these previous studies, elegant results from Cani et al. (88) and Turnbaugh et al. (89) demonstrated an association between the development of obesity and T2D, and an increase in the Firmicutes to Bacteroidetes ratio in the gut. This imbalance results in increased energy harvesting from food, as well as an increase in the transcription of genes controlling both lipogenesis in the liver and adipose tissue development (86, 89). Changes in the gut microbiota may also directly influence cells of both the innate (macrophages, dendritic cells, and innate lymphoid cells) and adaptive immune systems, thus contributing to development and maintenance of a state of chronic low-grade inflammation in obese individuals (75, 90).

Hydrolyzed carbohydrates and simple sugars can be fermented by gut microorganisms to short-chain fatty acids (SCFA), such as acetate, propionate, and butyrate. This sugar metabolism can result in an added 10% daily dietary energy to the host (91–93). Consumption of Western diets—low or normal in fiber contents and rich in fat and digestible sugars—can alter gut microbiota composition, impacting the production of SCFA and other gut-derived metabolites. Dysbiosis induced by fat- and sugar-rich diets is also associated with a thinner and less protective mucus layer, which results in increased gut permeability, chronic low-grade inflammation, and metabolic disorders (88, 94). Bacteria-derived SCFA have been shown to have anti-inflammatory properties attenuating the production of pro-inflammatory cytokines, such as TNF-α, IL-6, and IFN-γ. Thus, consumption of Western diets that results in decreased abundance of SCFA-producing bacteria could facilitate inflammatory responses in the gut (95).

## Food Additives That Alter Gut Microbiome: Emulsifiers and Sweeteners

Recent studies have demonstrated that the consumption of artificial sweeteners and dietary emulsifiers can alter the gut microbiota, resulting in intestinal disturbance and inflammation, favoring the development of the metabolic syndrome (27, 96).

Sweeteners such as high-fructose corn syrup and low-calorie or calorie-free sugar substitutes (sugar alcohols and artificial sweeteners) are among the most widely used food additives worldwide, regularly consumed by lean and obese individuals alike. A series of compounds (sucralose, aspartame, saccharin, steviol, Luo han guo extract, among others) are approved to be used as sweeteners in food, and are generally recognized as safe by the FDA. Cyclamates are banned in the United States but are still allowed in many other countries (97). Moreover, most of the commercial formulations of saccharin, sucralose, or aspartame comprise ~5% sweetener and ~95% glucose, functioning as a sugar source. Despite being considered safe and even beneficial owing to their low caloric content, pieces of evidence in various species, including humans, show that artificial sweeteners can be metabolically active (56, 98–104).

Pieces of evidence from both mice and human studies suggest that the consumption of artificial sweeteners is associated with increased glucose intolerance. This effect seems to result from alterations in the composition and function of the gut microbiota. In fact, several bacterial taxa that changed in response to sweetener consumption were previously associated with T2D development in humans (105, 106). For instance, it is reported that sweetener consumption increases the Bacteroides:Clostridiales ratio by increasing the former and decreasing the later. Indeed, the exposure of rats to Splenda (an artificial sweetener containing 1% sucralose) was associated with both increased weight gain and significant alterations in gut microbiota composition (23). In addition, the exposure to saccharin, sucralose, and especially aspartame-promoted alterations of the gut microbiota leading to an impaired glucose tolerance (24, 26). In fact, individuals consuming aspartame presented elevated fasting glucose and impaired insulin sensitivity, which were associated with gut dysbiosis (25).

Most artificial sweeteners are not chemically modified in the human gastrointestinal tract (107, 108) and, thus, directly encounter the intestinal microbiota. However, aspartame is a target for intestinal esterases and peptidases, which transforms aspartame into amino acids and methanol before reaching the colon (109). These products of aspartame metabolism should not have a significant influence on gut microbiota. Thus, the mechanisms of aspartame-induced dysbiosis remain unclear.

Currently, sucralose is widely used due to its taste (extremely similar to natural sugar) and providing no calories to the human body. Moreover, sucralose does not present the common and undesirable bitter aftertaste present in most of the artificial sweeteners. Therefore, sucralose has been considered a safe food additive mainly for the lack of any toxic effect and noeffect on metabolism (110). However, in a recent study, it was shown that sucralose consumption deeply impacts the gut microbiome composition increasing the release of pro-inflammatory mediators in mice. This effect was accompanied by an increase of inflammatory markers in the liver, such as MMP-2 and iNOS (26).

Saccharin is another sweetener strongly associated with the onset of metabolic diseases. Saccharin decreases the release of glucagon-like peptide-1 (GLP-1), an incretin hormone implicated in the regulation of various physiological processes, such as food intake, glycemic control, and cardiovascular protection (111, 112). Long-term cohort studies of artificial sweetener consumption reported increased risks of adverse outcomes, such as diabetes, cardiovascular diseases, and stroke, a pattern consistent with persistently reduced GLP-1 levels. On the other hand, Daly et al. (113) showed some prebiotic-like effects of saccharin/neohesperidin dihydrochalcone (SUCRAM^®^). Saccharin significantly increased gut abundance of Lactobacillus in piglets. Lactobacilli are the predominant lactic acid bacteria in the pig intestine and have well-described protective activity in the gastrointestinal tract, influencing gut immune system maturation and regulating intestinal inflammatory responses (113).

Emulsifiers are detergent-like molecules used by food industry as stabilizers for processed foods. Two commonly used emulsifiers are carboxymethylcellulose (CMC) and polysorbate-80 (P80). Toxicological and carcinogenic potential of P80 have been established and P80 is approved by the FDA for use in concentrations of up to 1%. CMC has not been extensively studied but it is used in various processed food at up to 2%. A recent study observed that, in mice, relatively low concentrations of these emulsifiers induced low-grade inflammation, obesity, and metabolic syndrome (27). These effects were associated with emulsifier-induced dysbiosis, bacterial translocation through the mucosal barrier, and increased pro-inflammatory potential. In fact, Roberts et al. (114) have shown that both emulsifiers can increase bacterial translocation across cultured epithelial layer (114). Moreover, bacterial translocation was associated with mucus loss and increased bacterial adherence after chronic exposure to dietary emulsifier (27).

Modest increases in body weight and fasting glycemia were observed in animals given doses as low as 0.1% CMC. Higher doses of 0.5% resulted in low-grade inflammation and increased adiposity. For P80, pieces of evidence of low-grade inflammation and increased adiposity were found in animals consuming amounts of as little as 0.1%. Higher concentrations of 0.5% resulted in a mild increase in fasting glycemia (27). These emulsifier-induced metabolic derangements were long-lasting since they could be observed even 6 weeks after emulsifier consumption withdraw (27).

Emulsifier-induced changes in gut microbiota play a role in driving inflammation and metabolic changes promoted by these food additives. They altered fecal levels of SCFA, including decreased levels of butyrate and bile acids (71, 115). In fact, emulsifiers induce gut barrier alterations and low-grade inflammation. They also result in significantly increased food intake (at least twice as high as the control mice), which drives the development of obesity by increasing fat mass and promoting metabolic alterations. Besides, excessive fat mass accumulation is associated with low-grade inflammation, which plays a crucial role in the onset of obesity and related metabolic disorders. Cani and Everard (116) reported that as the gut microbiota composition and the fecal SCFA profile are modified in emulsifier treated mice, such changes may also impact food intake. Growing evidence suggests that microbial products, for example, butyrate and propionate, may directly bind to specific G-protein-coupled receptors such as GPR41/43 (117), enriched in enteroendocrine L-cells. The stimulation of these receptors triggers the release of enteroendocrine peptides, such as GLP-1 and peptide YY that reduce food intake *via* a gut/brain axis.

In conclusion, emulsifiers and sweeteners, which are freely used by people controlling caloric intake as well as by obese and patients with diabetes, present a verified effect on gut microbiota. This effect results in dysbiosis that normally is associated with the development of insulin resistance and to weight gain. Therefore, these additives should not be considered as innocuous and must have their use controlled and labeled with regard to possible undesirable effects.

## Association of Food Additives: Carrageenan

Carrageenan is a hydrocolloid extracted from seaweeds and widely used by food industry as thickener and stabilizer. Due to its high molecular weight, it is assumed that carrageenan is not absorbed in the intestine and, thus, can be safely consumed (118). In fact, orally administered carrageenan can be recovered in feces in substantial amounts (119). Recovered carrageenan behaves similarly to the original in chromatographic assays, suggesting that it is stable at low pH and resistant to bacterial degradation, not being substantially altered or metabolized in the gastrointestinal tract (119).

However, recent studies demonstrate that oral carrageenan administration can lead to insulin resistance. This suggests that either there is a mechanism of immune cell activation in the gut or that the absorption of even trace amounts of carrageenan is sufficient to trigger pro-inflammatory responses systemically.

Carrageenan has been widely used as an inducer of inflammation and inflammatory pain (120). Its pro-inflammatory potential results from TLR4 activation, an innate immune receptor also involved in obesity and insulin resistance (12). This renders carrageenan as a potential to trigger for cellular processes that would contribute to the establishment of metabolic syndrome. For example, intratumoral carrageenan administration was recently reported to inhibit tumor progression by shifting macrophages toward a M1 phenotype (121).

In that sense, recent studies report the effect of administering carrageenan in water on metabolic parameters in mice. It was first shown that carrageenan consumption—in doses equivalent to those in foodstuff—leads to glucose intolerance *via* increased IRS-1 serine phosphorylation and interference with insulin-triggered PI3K–Akt pathway (34). This was also observed *in vitro*, using the human hepatocyte cell line HepG2, suggesting that a similar mechanism may be operating in human obesity. Further studies by the same group demonstrated that interference with insulin signaling by carrageenan was triggered *via* a signaling pathway involving TLR4, Bcl10, and mitochondrial ROS, which was further sustained by GRB10 (122). GRB10 is an adaptor protein that associates with and inhibits IRS-1 and was upregulated by carrageenan exposure both *in vitro* and *in vivo* (122).

Interestingly, carrageenan consumption exacerbated glucose intolerance and dyslipidemia induced by high-fat diet feeding (35). Of note, carrageenan did not show any effect on lipid profile of mice fed a normal diet, but induced a substantial increase in non-HDL cholesterol levels. This suggests that carrageenan consumption, especially in association with fat rich food (as seen in industrialized foods), may predispose individuals to develop atherosclerosis and increase cardiovascular risk.

The safety of carrageenan consumption is still a matter of debate. Arguments in favor of its safety rely on toxicological studies showing no significant intestinal absorption after oral administration. In these studies, there was no evidence of carrageenan traces in the liver or cells of the gastrointestinal tract of non-human primates and rats (123, 124), although the analytical method applied were not very sensitive. By using a more sensitive method, administering radiolabeled carrageenan, it was observed the presence of absorbed carrageenan in cecal lymph node, Peyer’s patches, and the intestinal wall, mainly associated with macrophages (125). Other authors showed the presence of macrophage-borne carrageenan in intestinal lamina propria and the liver, suggesting that sampling of intestinal content by mucosal macrophages may provide a route for carrageenan entry (126, 127).

Despite the lack of consensus on whether carrageenan is absorbed or not, its pro-inflammatory activity is undeniable. Active absorption or passive entry of even trace amounts of carrageenan could have significant effects on metabolic homeostasis, especially with chronic exposure. Given that a high-fat diet or food additives such as emulsifiers were shown to interfere with intestinal mucosal permeability, it is important to reconsider the toxicological properties of carrageenan. For example, the addition of carrageenan (as a thickener) to foods rich in fat could facilitate the extravasation of carrageenan due to high-fat-induced intestinal permeability. Likewise, association of carrageenan with emulsifiers (as seen in many processed foods) would facilitate carrageenan absorption after disruption of the epithelial intestinal barrier. Therefore, in light of these new data showing that the intestinal barrier homeostasis may be disrupted by dietary components, it becomes necessary to reassess the toxicological properties of this widely used food additive.

## Conclusion

Modern societies have witnessed an alarming increase in cases of obesity, T2D, and other comorbidities associated with poor eating habits. In addition, the incorporation of processed and industrialized food into everyday eating correlates with this phenomenon. In an attempt to tackle that threat, much attention has been paid to the amount of calories present in food. Specifically, the amount of fat and simple sugar is considered the main villains of modern processed food. Although the detrimental effects of fat and sugar are undeniable, industrialized food is composed of many other substances of which the metabolic effects are usually ignored. Emulsifiers, thickeners, artificial sweeteners, and preservatives may have direct and indirect effects on cells of the immune system, contributing to metabolic dysregulation. Moreover, safety evaluation and estimates of food additives toxicity are usually based on tests of single compounds, thus underestimating the effects of associating two or more additives. Therefore, policies that aim at curbing the evolution of obesity must take into account not only calorie content of food but also all the other components and their effects. Whenever possible, people should avoid ready-to-serve, additive rich, processed food and prefer healthier food choices or home-cooked meals.

## Author Contributions

HN conceived the text and wrote the manuscript. PA, LG, JL, PZ, and MS-P wrote the manuscript.

## Conflict of Interest Statement

The authors declare that the research was conducted in the absence of any commercial or financial relationships that could be construed as a potential conflict of interest.
